# ﻿A new combination and synonym in *Bupleurum* (Apiaceae, Apioideae), based on morphological, molecular and cytological evidence

**DOI:** 10.3897/phytokeys.239.116877

**Published:** 2024-03-21

**Authors:** Li-Hua Wang, Shuo Li, De-Ning Zhang, Quan-Ru Liu

**Affiliations:** 1 Key Laboratory of Biodiversity Science and Ecological Engineering, Ministry of Education, College of Life Sciences, Beijing Normal University, Beijing 100875, China2 Qinghai Shanshui Natural Resources Survey, Planning and Design Institute, Xining 810008, Qinghai, China Beijing Normal University Beijing China; 2 Qinghai Shanshui Natural Resources Survey, Planning and Design Institute, Xining 810008, Qinghai, China Qinghai Shanshui Natural Resources Survey, Planning and Design Institute Xining China

**Keywords:** Apiaceae, chloroplast genome, chromosome counts, new combination, synonymy

## Abstract

Specimen examinations and field observations revealed that Bupleurumsmithiivar.parvifolium was distinctly different from B.smithiivar.smithii in umbel, leaf, and fruit morphology, but was very similar to B.commelynoideumvar.flaviflorum. Based on these morphological evidences, the present study re-examined the taxonomic status of these taxa through morphological, cytological, and phylogenetic analyses. The results showed distinguishable features in the width of middle leaves and bracteoles of B.smithiivar.parvifolium compared to B.smithiivar.smithii. Morphological variation between B.smithiivar.parvifolium and B.commelynoideumvar.flaviflorum was continuous and overlapping. Notably, the chromosome number of B.smithiivar.parvifolium was 2n = 14 (x = 7), consistent with B.commelynoideumvar.flaviflorum, whereas B.smithiivar.smithii was 2n = 64 (x = 8). Additionally, phylogenetic analyses revealed B.commelynoideumvar.flaviflorum nested within B.smithiivar.parvifolium, and that both were distant from the B.smithiivar.smithii and B.commelynoideumvar.commelynoideum. Based on the evidence above, the differences between B.smithiivar.parvifolium and B.smithiivar.smithii extend beyond the level of intraspecific variation, and B.commelynoideumvar.flaviflorum is considered to be identical with B.smithiivar.parvifolium. Hence. A new combination and status, *B.parvifolium* (Shan & Y.Li) Q.R.Liu & L.H.Wang, **comb. et stat. nov.**, is proposed. Furthermore, B.commelynoideumvar.flaviflorum should be treated as a synonym of *B.parvifolium*.

## ﻿Introduction

The genus *Bupleurum* (Apiaceae, Apioideae), comprising 180–220 species (Sheh and Watson 2005; https://wfoplantlist.org/plant-list/), is widely distributed in the North Temperate Zone of Eurasia and is utilized in traditional herbal medicines in China, East Asia, and North Africa (Teng et al. 2023). Phylogenetic studies supported the *Bupleurum* as a basal clade within the subfamily Apioideae and categorized this morphologically unusual genus as the monotypic tribe Bupleureae Spreng. (Downie et al. 2000). 42 species and 16 varieties are recorded in the “Flora of China”, widely distributed in the NE, NW, and SW of China, and approximately 22 taxa (included varieties) are endemic (Sheh and Watson 2005). Due to the high morphological variability of *Bupleurum* and the quantitative traits used for interspecific identification, species identification is challenging and the taxonomic status of some taxa is uncertain. It is necessary to conduct comprehensive studies by integrating multiple lines of evidence (e.g., cytology, phylogenetic, and biogeography) to clarify the taxonomic status of taxa in doubt.

*Bupleurumsmithii* Wolff was classified into three varieties: B.smithiivar.smithii, B.smithiivar.parvifolium Shan & Y.Li, and B.smithiivar.auriculatum Shan & Y.Li, based on leaf morphology (Shan and Li 1974). After reviewing the specimens and field surveys, it was found that the B.smithiivar.parvifolium was dwarfed, the leaves became narrower and smaller from B.smithiivar.smithii. Bupleurumsmithiivar.smithii is a widely distributed species in Northeast China, thriving above 1800 m. The species was initially described based on collections from Xiaowutai Mountain, situated within the Taihang Mountains. On the other hand, B.smithiivar.parvifolium is prevalent in the grasslands of the Qinghai–Tibetan Plateau, with type specimens collected from Wushaoling, belonging to the Qilian Mountains (Shan and Li 1974). The distribution areas of these varieties are significantly separated, showing clear discontinuity. However, existing studies on phylogenetic analyses suggested that B.smithiivar.smithii was more closely related to B.smithiivar.parvifolium and more distantly related to B.smithiivar.auriculatum (Wang et al. 2011). Phylogenetic analyses and morphological observations conflicted. Morphologically and geographically, *B.smithii* is more similar to B.smithiivar.auriculatum, instead of B.smithiivar.parvifolium. This precisely suggests that the taxonomic status of B.smithiivar.parvifolium needs to be researched further.

Shan and Li (1974) formally described B.commelynoideumvar.flaviflorum Shan & Y.Li based on type specimens collected from Min County, Gansu province. It can be differentiated from *B.commelynoideum* by its flower color and the shape of bracteoles: B.commelynoideumvar.flaviflorum displays yellow flowers and narrowly ovate bracteoles, while B.commelynoideumvar.commelynoideum exhibits purple flowers and broadly ovate bracteoles. Shan and Li (1974) also noted B.commelynoideumvar.flaviflorum affinity to *B.smithii*, particularly B.smithiivar.parvifolium, which features a short, slightly creeping stem, basal leaves that are not long-acuminate, and bracteoles that are occasionally reduced to five. These traits are often underdetermined and closely related to the environment. Regarding distribution patterns, B.commelynoideumvar.flaviflorum was predominantly found in Southwest China (Gansu, Qinghai, and Sichuan), while B.smithiivar.parvifolium was distributed in Northwest China (Gansu, Qinghai, Ningxia, Xizang, and Sichuan), with overlapping occurrences in Northwest Sichuan and South Gansu. A molecular study based on ITS, *trnH-psbA*, and *matK* by Wang et al. (2011) demonstrated B.commelynoideumvar.flaviflorum nested within B.smithiivar.parvifolium and B.smithiivar.smithii. Consequently, they proposed reclassifying B.commelynoideumvar.flaviflorum as a variety of *B.smithii*, naming it B.smithiivar.flaviflorum (Shan & Y.Li) X.J.He & C.B.Wang. More recently, Ma (2015a) suggested that there is no interrupted morphological difference between B.commelynoideumvar.flaviflorum and B.smithiivar.parvifolium, implying that they might be the same species.

Upon a thorough examination of the type specimens, it was discovered that the isotype specimen of B.commelynoideumvar.flaviflorum was identified as B.commelynoideumvar.flaviflorum (WUK0423353) and B.smithiivar.parvifolium (WUK0033909). Such instances are common during specimen reviews, highlighting the need for meticulous morphological and phylogenetic analyses to elucidate the taxonomic placement and phylogenetic position of these taxa.

## ﻿Materials and methods

### ﻿Morphological studies

Our study involved the examination of collections and digital images of B.smithiivar.smithii, B.smithiivar.parvifolium, B.smithiivar.auriculatum, B.commelynoideumvar.flaviflorum, B.commelynoideumvar.commelynoideum from the Chinese Virtual Herbarium (http://www.cvh.ac.cn/) and the Global Biodiversity Information Facility (https://www.gbif.org/). The images were sourced from specimens deposited at BJFC, BM, BNU, CDBI, HNWP, HSIB, KUN, NAS, P, PE, PEY, SZ, and WUK. The specimens with well-preserved leaves and flowers were selected, covering all districts of the distribution area. A total of 43 specimens of B.smithiivar.smithii, 50 of B.smithiivar.parvifolium, 29 of B.commelynoideumvar.commelynoideum, and 39 of B.commelynoideumvar.flaviflorum were examined, including 55 sheets of specimens collected by our team (Suppl. material 1). Given the substantial variation in leaf shape with the growth period, and the significant differences between basal, middle, and upper leaves, we selected 3 basal leaves, 2 middle leaves, and 2 upper leaves for each specimen. A total of 37 morphological characters were measured (Suppl. material 1), and after Principal Component Analysis (PCA), 16 traits were chosen for subsequent analyses, including the length, width, and ratio of length/width of basal leaves, as well as the number, length, and width of bracteoles (Suppl. material 2).

The examination of characters was conducted using ImageJ (Rasband 1997). PCA and Cluster analysis were performed in R, utilizing the factoextra package. Factoextra relies on ggplot2 (Wickham 2009), FactoMineR (Le et al. 2008), and cluster (Maechler et al. 2016) for visualization and analysis.

### ﻿Cytology

The materials used in the cytological studies were sourced from Xiaowutai Mountain (for B.smithiivar.smithii), Qilian Mountain (for B.smithiivar.parvifolium), Luya Mountain (for B.smithiivar.auriculatum) and Min Mountain (for B.commelynoideumvar.flaviflorum). The voucher specimens are detailed in Table 1, asterisks. Chromosome counts were carried out through acid digestion and wall removal hypotonic procedures, adapted from Li et al. (2021). Each sample underwent three repetitions in the experimental protocol.

**Table 1.** Voucher information and GenBank accession numbers of newly sequenced plastome sequences, asterisks for cytology.

Taxa	Location	Voucher information	Accession
*B.baimaense*	Deqincountry, Yunnan, China	YNNU-19-302 (KUN)	OR778864
B.commelynoideumvar.commelynoideum	Kangding, Sichuan, China	BNU2023WLH0174 (BNU)	OR778865
B.smithiivar.smithii *[1]	Xiaowutai Mountain, Hebei, China	BNU2020DT007 (BNU)	OR387522
B.smithiivar.smithii [2]	Dongling Mountain, Hebei, China	DL023-3 (BNU)	OR811239
B.smithiivar.auriculatum*	Luya Mountain, Shanxi, China	BNU2021SX017 (BNU)	OR811240
B.commelynoideumvar.flaviflorum*	Min Mountain, Gansu, China	BNU2023WLH190 (BNU)	OR778866
B.smithiivar.parvifolium [1]	Hualong County, Qinghai, China	BNU2022WLH061 (BNU)	OR778870
B.smithiivar.parvifolium [2]	Haiyan County, Qinghai, China	ZZU2021QH004 (BNU)	OR778869
B.smithiivar.parvifolium* [3]	Tianjun county, Qinghai, China	BNU2022WLH030 (BNU)	OR778871

### ﻿Plant material, DNA extraction, sequencing, assembly and annotation

In this study, the whole chloroplast (cp) genome of *Bupleurum* was sequenced to investigate its phylogeny and evolution. Fresh leaves from nine *Bupleurum* specimens were field-collected and rapidly desiccated using Silica Gel. Voucher specimens were deposited at Herbarium of Beijing Normal University (BNU), and listed in Table 1. Genomic DNA extraction was extracted using the HP Plant DNA Kit D2485-02 kit (Omega Bio-Tek), with Beijing Novogene Corporation conducting assessments of DNA quantity and quality. The Illumina HiSeq X Ten sequencing platform was employed, generating approximately 10 GB for each sample. The chloroplast genome was assembled by GetOrganelle (Jin et al. 2020). PGA was used to annotate (Qu et al. 2019), with the cp genome of *B.yinchowense* (MT075711) and *B.sikangense* (NC056803) as references. The cp genome has been submitted to the NCBI GenBank database (www.ncbi.nlm.nih.gov). Additionally, 18 genome sequences were downloaded from NCBI (Appendix 1), and 2 *Pleurospermum* were selected as outgroups.

### ﻿Phylogenetic analyses

Phylogenetic analyses were conducted using the maximum likelihood (ML) and Bayesian inference (BI) methods with IQ-TREE (Minh et al. 2020) and MrBayes (Ronquist et al. 2012), respectively. A total of 27 sequences were aligned using the online version of MAFFT (https://mafft.cbrc.jp/alignment/server/index.html) (Katoh et al. 2019). The ModelFinder module (Kalyaanamoorthy et al. 2017) in PhyloSuite (Zhang et al. 2020) determined the best-fit model of nucleotide substitutions. The nucleotide substitution model for the ML tree was TVM+R2+F, and standard bootstrap (BS) replicates of 1000 were performed, with results deemed reliable at BS ≥70%. For the BI tree, the best model was GTR+F+I+G4, and the analysis parameters were set as follows: mcmcp ngen = 2000000, printfreq = 10000, nchains = 4, and burninfrac = 25%. Reliable results were considered when the Posterior probability (PP) was ≥0.95. The effective sample size (ESS) (>200) was confirmed using Tracer v1.7 (Rambaut et al. 2018).

## ﻿Results and discussion

### ﻿Morphological studies

**Figure 1.**
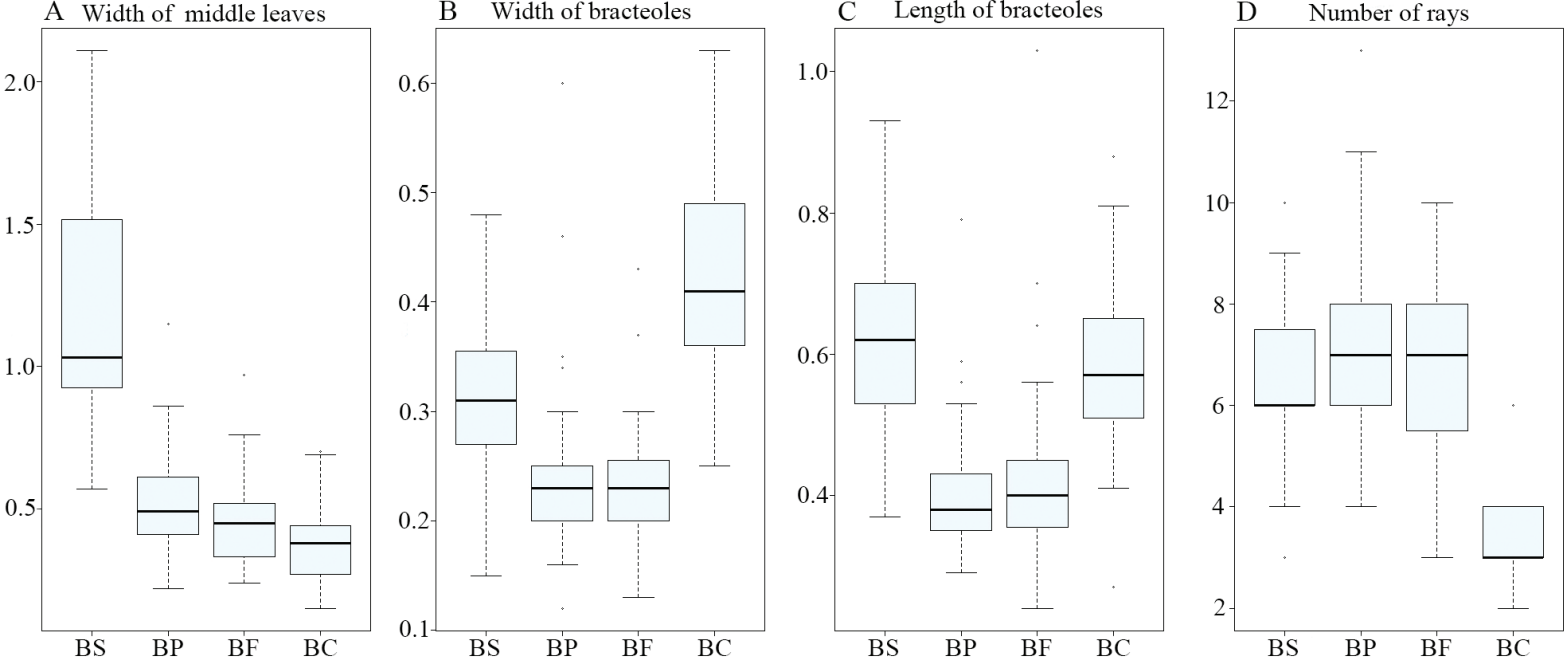
Comparison of the characters **A** width of middle leaves **B** width of bracteoles **C** length of bracteoles **D** number of rays. **BS** = Bupleurumsmithiivar.smithii**BP** = B.smithiivar.parvifolium**BF** = B.commelynoideumvar.flaviflorum**BC** = B.commelynoideumvar.commelynoideum.

**Figure 2.**
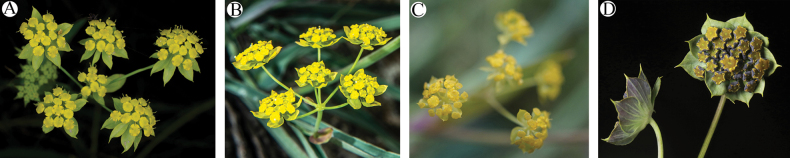
Umbel and bract **A**Bupleurumsmithiivar.smithii**B**B.smithiivar.parvifolium**C**B.commelynoideumvar.flaviflorum**D**B.commelynoideumvar.commelynoideum.

**Figure 3.**
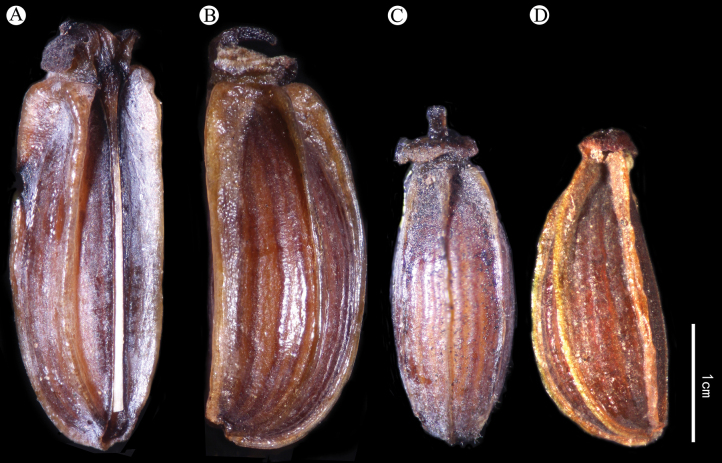
Fruit morphology **A**Bupleurumsmithiivar.smithii**B**B.smithiivar.auriculatum**C**B.smithiivar.parvifolium**D**B.commelynoideumvar.flaviflorum

**Figure 4.**
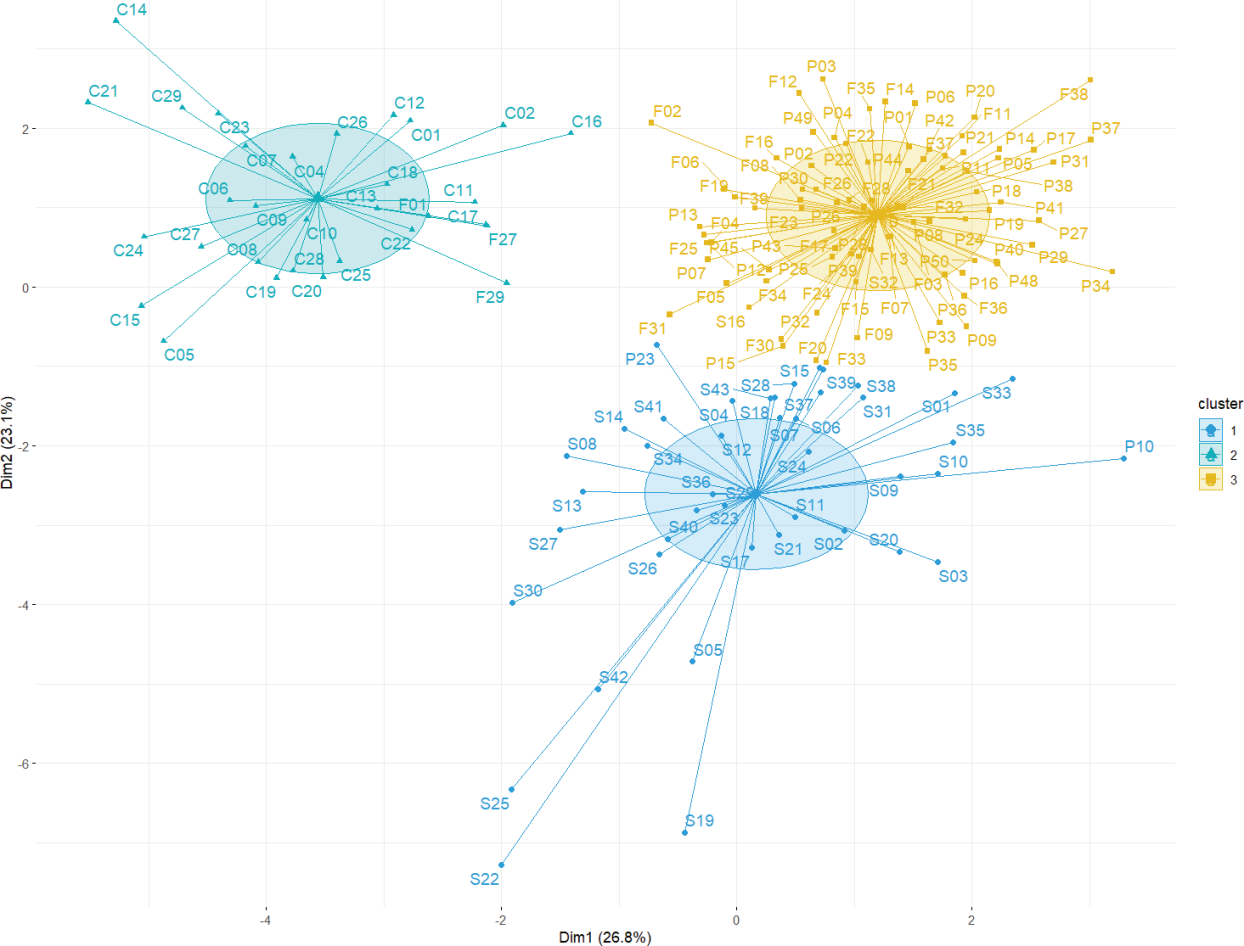
Cluster plot for the morphological variations among four taxa. **C** = Bupleurumcommelynoideumvar.commelynoideum**F** = B.commelynoideumvar.flaviflorum**P** = B.smithiivar.parvifolium**S** = B.smithiivar.smithii.

The Principal Component Analysis (PCA) revealed that traits such as width of middle leaves, width of bracteoles, length of bracteoles, and the number of rays were effective for interspecific classification (Fig. 1). Firstly, the number of rays (Fig. 1D) and petal color (Fig. 2) emerged as distinguishing features for B.commelynoideumvar.commelynoideum compared to other taxa. Bupleurumcommelynoideumvar.commelynoideum typically exhibited 3–4 rays and purplish-red petals, while the other three taxa had more than 5 rays and yellow petals. Secondly, the width of middle leaves (Fig. 1A), length and width of bracteoles (Fig. 1B and 1C) were effective in differentiating B.smithiivar.smithii from B.smithiivar.parvifolium and B.commelynoideumvar.flaviflorum. Additionally, the fruits of B.smithiivar.smithii and B.smithiivar.auriculatum were longer, 3–3.5 mm, while those of B.smithiivar.parvifolium and B.commelynoideumvar.flaviflorum measured 2–2.5 mm (Fig. 3). Notably, all traits in Fig. 1 showed no discontinuity between B.smithiivar.parvifolium and B.commelynoideumvar.flaviflorum, and none could effectively differentiate between the two taxa. The Cluster plot illustrated that B.smithiivar.smithii and B.commelynoideumvar.commelynoideum formed distinct groups, while B.smithiivar.parvifolium and B.commelynoideumvar.flaviflorum clustered together comprehensively. Only individual specimens from these varieties exhibited clustering within other groups (Fig. 4). Morphology is compared and described in Table 2.

**Table 2.** Comparison of morphological characteristics and geographic distribution.

Morphology	B.smithiivar.smithii	B.smithiivar.parvifolium	B.commelynoideumvar.flaviflorum	B.commelynoideumvar.commelynoideum
**Basal leaves**	7–15 × 0.8–1.5 cm	6–8 × 0.3–0.7 cm	6–8 × 0.3–0.5 cm	8–15 × 0.25–0.4 cm
**Middle stem leaves**	7–13 × 0.8–1.5 cm	4–7 × 0.4–0.7 cm	6–9 × 0.3–0.5 cm	8–11 × 0.25–0.4 cm
**Umbels**	Numerous	Numerous	Numerous	Single
**Rays**	4–9	4–9	4–9	3–4 (6)
**Bracteole**	(6)7–9, 5–7 × 3–4.5 mm	(5) 6–7, 3–4.5 × 2–2.5 mm	(5) 6–7, 3–5 × 2–2.5 mm	7–9, 5–6.5 × 3–5 mm
**Petal color**	Yellow or abaxially purplewish-tinged	Yellow or abaxially purplewish-tinged	Yellow or abaxially purplewish-tinged	Purple or yellowish-tinged
**Fruit**	Rectangular, 3.0–3.6 × 1.2–1.4	Ovoid, 1.9–2.5 × 1.1–1.3	Ovoid, 2.0–2.4 × 1.1–1.3	Tapered, 2–2.5 × 1.5
**Vittae in each furrow**	4	6	6	4
**Vittae on commissure**	3	3	3	3
**Distribution**	Shanxi, Hebei, Beijing and N Henan	E Qinghai, Gansu, Ningxia, E Xizang, and NW Sichuan	S Gansu, S Qinghai, and W Sichuan.	W Sichuan, Xizang and NW Yunnan

PCA and box plots showed intermittent distinctions in leaf shape, bracteoles, and fruit size between B.smithiivar.smithii and B.smithiivar.parvifolium, providing reliable evidence for interspecific differentiation. Cluster diagram results corroborated the cohesion of B.smithiivar.parvifolium and B.commelynoideumvar.flaviflorum into a single group, providing robust support for considering them as the same taxon. Box plots visually depicted continuous and overlapping variations between these two taxa. Ma (2015a) concluded that B.smithiivar.parvifolium and B.commelynoideumvar.flaviflorum do not have intermittent morphological differences, and B.commelynoideumvar.flaviflorum may be a synonym of B.smithiivar.parvifolium, which is formally proposed herein. Morphological observations support that B.smithiivar.parvifolium is a separate species and B.commelynoideumvar.flaviflorum is the same entity as B.smithiivar.parvifolium, and the former should be treated as a synonym of the latter.

### ﻿Cytology

**Figure 5.**
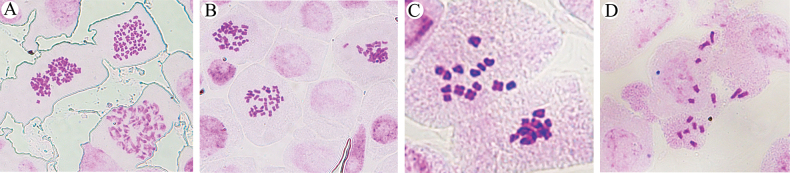
Metaphase chromosomes **A**Bupleurumsmithiivar.smithii**B**B.smithiivar.auriculatum**C**B.smithiivar.parvifolium**D**B.commelynoideumvar.flaviflorum.

Cytological analysis reveals that the chromosome number of B.smithiivar.parvifolium is 2n = 14 (x = 7) (Fig. 5B), consistent with B.commelynoideumvar.flaviflorum (Fig. 5C). In contrast, *B.smithii* exhibited a chromosome number of 2n = 64, and that of B.smithiivar.auriculatum is 2n = 32 (Fig. 5A). These findings align with previous reports, as documented by Liang et al. (2013).

Chromosome base diversity is high within the genus *Bupleurum*, including several cases with x = 4, 5, 6, 7, 8, 11, and 13 (Neves and Watson 2004; Wang et al. 2011; Liang et al. 2013). Different ploidy often occurs within a species, but the chromosome base is mostly the same. It has been found that *B.commelynoideum* exhibited complex variations in both chromosome ploidy (2x, 4x, 6x) and basic number (x = 5, x = 6) (Liang et al. 2013; Wang et al. 2011). Then, Ma et al (2015b) combined cytological and phylogenetic evidence to confirm the existence of at least three cryptic species within *B.commelynoideum*. The importance of accurate chromosome number documentation cannot be overstated, particularly given instances of misidentification. Notably, *B.smithii* was initially reported with a chromosome of 2n = 12 (Alexeeva et al. 2000). However, the voucher specimen was collected from Songpan, Sichuan Province. However, this region is not the distribution area of *B.smithii*. It is hypothesized that it may be a misidentification of other taxa, but there are no voucher specimens. To address this, we revisited the type locality, collecting specimens that underwent meticulous comparison with the type specimen, and confirming a chromosome count of 64 for B.smithiivar.smithii. This represents the highest reported chromosome number in the genus, presumed to be an octoploid with a chromosome base of 8, akin to *B.sibiricum* (Chin et al. 1989; Jiang et al. 1994). The chromosome number of B.smithiivar.auriculatum is 32, presumably tetraploid. Chromosome ploidy was not the same in B.smithiivar.smithii and B.smithiivar.auriculatum, but the base number was the same. Therefore, B.smithiivar.auriculatum will still be suitable as a variant of *B.smithii* from chromosome analysis.

In recent years, researchers have gradually emphasized the role of chromosomes in species delimitation. For example, *B.komarovianum* was once treated as a variety of *B.chinense* (Liaoning Forestry Soil Research Institute 1977). Wang et al. (2011) suggested it should still be a separate species based on chromosomal evidence and morphology, *B.komarovianum* was 2n = 2x = 8 (*B.chinense* was 2n = 2x = 12) and the stem of *B.komarovianum* was hollow (*B.chinense* was with pith). The chromosome information for both B.smithiivar.parvifolium and B.commelynoideumvar.flaviflorum are diploid with a total of 14 chromosomes. The number and base of chromosomes support that the two taxa are the same entity, and they are different from B.commelynoideumvar.commelynoideum and B.smithiivar.smithii.

### ﻿Phylogenetic analyses

**Figure 6.**
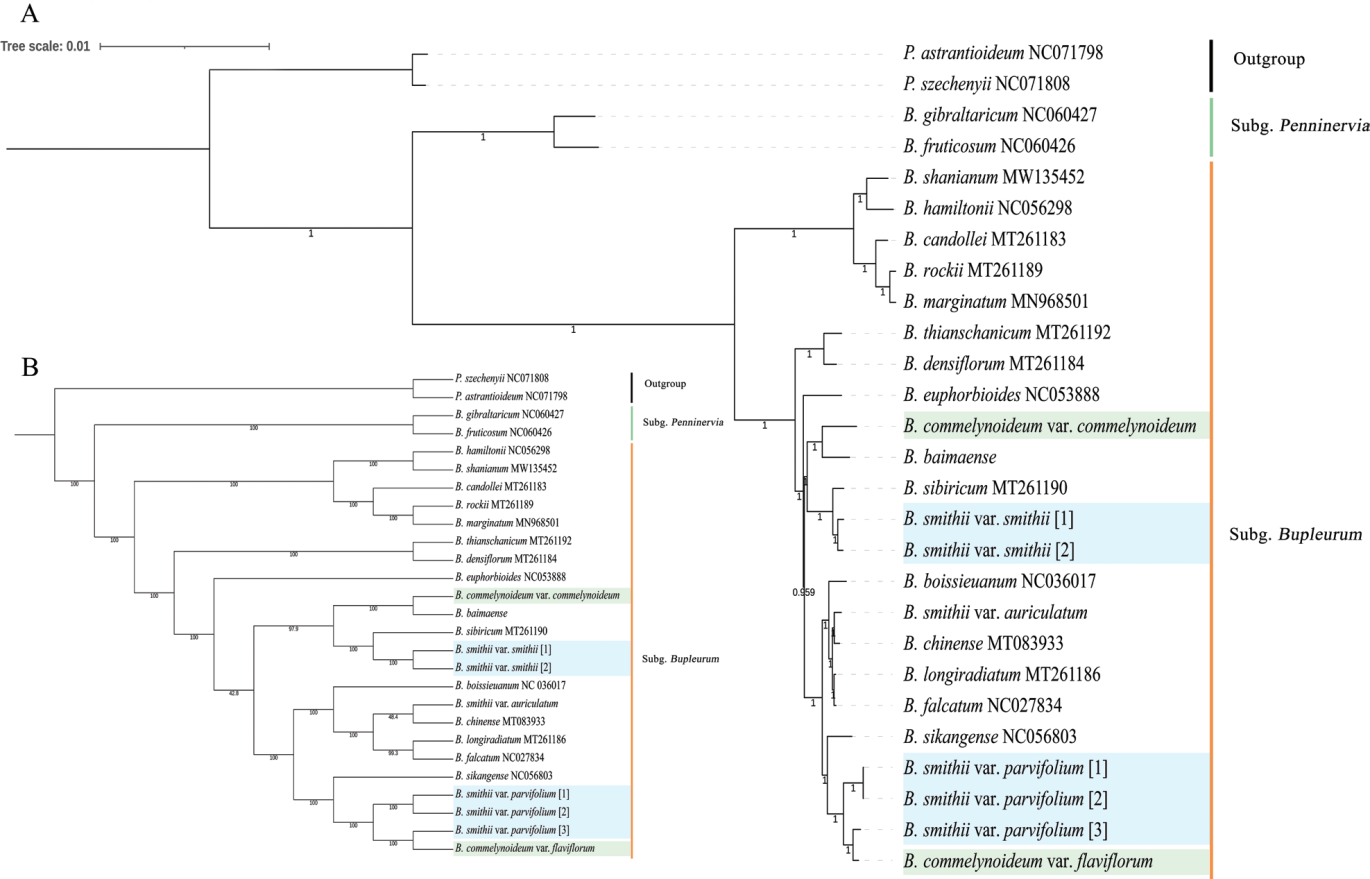
The phylogenetic tree was inferred from chloroplast genomes **A**BI analyses **B**ML analyses

The phylogenetic analysis based on chloroplast genome sequences reveals a consistent topology between the maximum likelihood (ML) tree and the Bayesian inference (BI) tree. The genus *Bupleurum* was divided into two clades with high support (BS = 100%, PP = 1), with all Chinese Bupleurum species belonging to Subg. Bupleurum. Bupleurumcommelynoideumvar.flaviflorum was nested within B.smithiivar.parvifolium (BS = 100%, PP = 1), forming an individual clade. This clade was further related to *B.sikangense* X.J.He & C.B.Wang (Fig. 6), and all these taxa were distantly related to B.smithiivar.smithii and B.commelynoideumvar.commelynoideum. *Bupleurumsmithii* was closely related to *B.sibiricum* Vest ex Spreng, while *B.commelynoideum* was closely related to *B.baimaense* X.G. Ma & X.J. He (BS = 100%, PP = 1).

In this study, the reconstruction of the phylogenetic tree utilizing the chloroplast genome yielded results consistent with the topological structure presented by Wang et al. (2011). When compared to the phylogenetic tree, which employed nuclear ribosomal internal transcribed spacer, *trnH-psbA*, and *matK*, the reconstructed tree based on the chloroplast genome exhibited greater support and resulted in changes in the phylogenetic positions of several species. In his study, B.smithiivar.parvifolium and B.commelynoideumvar.flaviflorum were nested together, forming a sister group to B.smithiivar.smithii and *B.pusillum*. However, it is noted that the material of B.smithiivar.smithii was collected in Minhe, Qinghai, the primary distribution zone of B.smithiivar.parvifolium, not within the range of B.smithiivar.smithii. This discrepancy raises speculation that Wang might have misidentified B.smithiivar.parvifolium as B.smithiivar.smithii. Conversely, in his paper B.smithiivar.auriculatum was collected from Wutai Mountain, which closely aligns with the type location of B.smithiivar.smithii. Both studies, using different markers, indicate a close relationship between B.commelynoideumvar.flaviflorum and B.smithiivar.parvifolium. However, the systematic position of B.smithiivar.auriculatum is uncertain, and the affinity between B.smithiivar.auriculatum and B.smithiivar.smithii has to be determined by more molecular markers in the follow-up.

### ﻿Distribution

**Figure 7.**
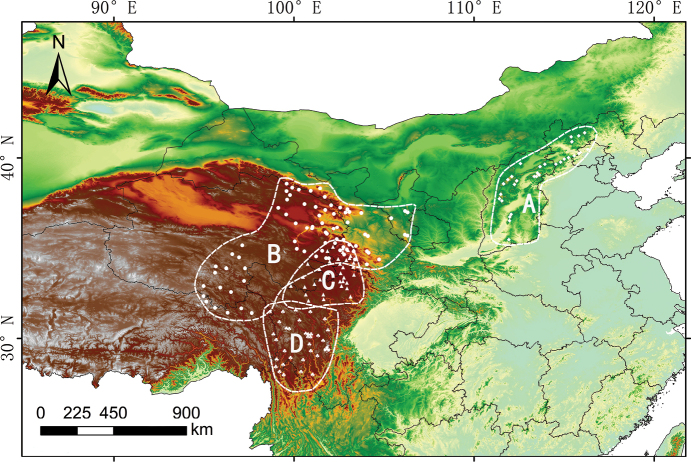
Distribution area based on specimen records and our field investigation **A**Bupleurumsmithiivar.smithii**B**B.smithiivar.parvifolium**C**B.commelynoideumvar.flaviflorum**D**B.commelynoideumvar.commelynoideum

In terms of distribution patterns, B.smithiivar.parvifolium is a widespread taxon mainly in northwest China, including Gansu, Ningxia, Qinghai, eastern Xizang, and western Sichuan. On the other hand, B.commelynoideumvar.flaviflorum is distributed in SW China, including S Gansu, S Qinghai, and W Sichuan. The distribution of these two taxa overlaps in northwest Sichuan and southern Gansu, precisely where the type specimens of B.commelynoideumvar.flaviflorum were collected. In contrast, B.smithiivar.smithii primarily occur in the Taihang Mountains and Yanshan Mountains in E China. *Bupleurumcommelynoideum* is a species that occurs throughout the Hengduan Mountains region. The distribution areas of B.smithiivar.parvifolium and B.smithiivar.smithii are separated by the Qinling Mountains and the Loess Plateau (Fig. 7).

### ﻿Taxonomic treatment

#### ﻿ Bupleurum parvifolium

Taxon classificationPlantaeApialesApiaceae

(Shan & Y.Li) Q.R.Liu & L.H.Wang, comb. et stat. nov.

F42A48A0-8E5B-573B-9561-E3C3BC8F22B8

urn:lsid:ipni.org:names:77338773-1

≡ B.smithiivar.parvifolium Shan & Y.Li, Acta Phytotax. Sin. 12(3): 273. 1974. Type. CHINA. Gansu: Tianzhuxian, Wushao Mountain ca. 2800 m, 22. 07. 1959, *Y.Q. He 4267* (Holotype: WUK0389736!). Basionym.
= B.commelynoideumvar.flaviflorum Shan & Y.Li, Acta Phytotax. Sin. 12(3): 276. 1974. syn. nov. ≡ B.smithiivar.flaviflorum (Shan & Y.Li) X.J.He & C.B.Wang J. Syst. Evol. 49 (6): 586. 2011.Type. CHINA. Gansu: Min County, Min Mountain, ca. 3500 m, 10. 08. 1937, *T.P. Wang 7535* (Holotype: PE00935517! Isotypes: WUK0033909! WUK0423353!).

##### Diagnosis.

*Bupleurumparvifolium* is morphologically similar to *B.smithii*, can be distinguished from the latter by its cauline leaves sessile, 4–9 × 0.3–0.7 cm; bracteoles 6–9, ovate or broad-ovate, 3–5 × 2–2.5 mm; fruit ovoid, brown, 2.0–2.5 × 1.1–1.3 mm; vittae 3 in each furrow, 6 on commissure.

##### Description.

**Figure 8.**
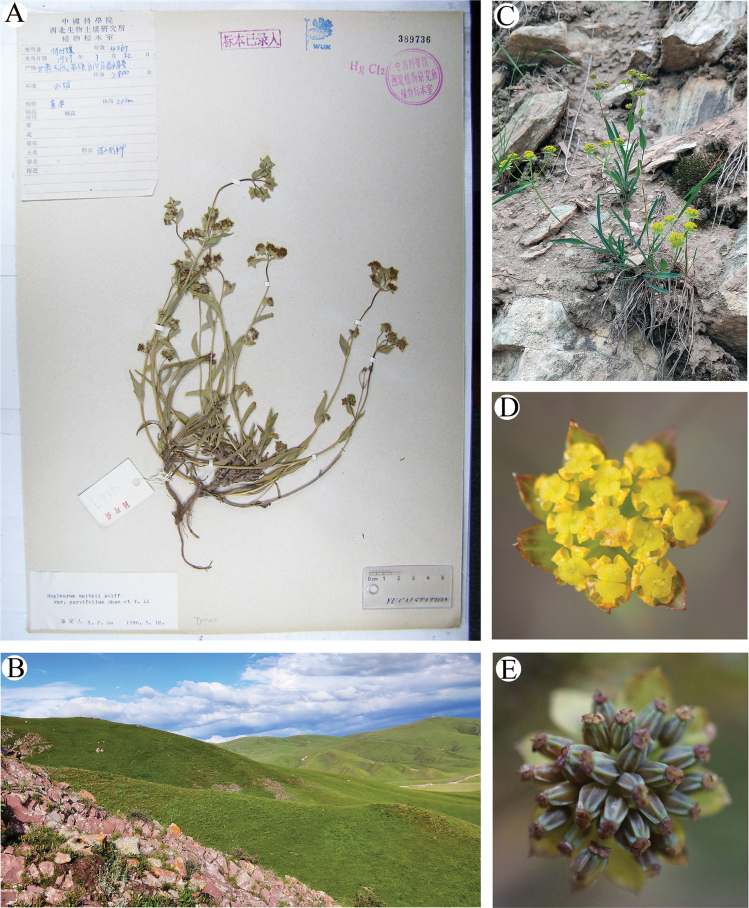
*Bupleurumparvifolium***A** holotype **B** habitat **C** plant **D** flowers **E** fruits.

Plant 15–40 cm, perennial. Rhizome brown, usually branched. Stems many, tufted, base without fibrous remnant sheaths. Basal leaves narrowly lanceolate, 6–8 × 0.3–0.7 cm, thickly papery, base tapered into petiole, not embracing. Cauline leaves sessile, 4–9 × 0.3–0.7 cm. Apical leaf long-ovate, 1.5–7.5 × 1–1.7 cm, base rounded, sometimes auriculate, clasping, apex acuminate. Bracts 0 or 1–2, broadly ovate, 7–18 × 4–11 mm, unequal; rays 4–9, 0.5–4 cm, unequal, angled; bracteoles 6–9, ovate or broad-ovate, 3–5 × 2–2.5 mm, equal, acute, apiculate, slightly exceeding flowers; umbellules 0.8–1.1 cm across. Petals yellow, occasionally abaxially purplish-red. Stylopodium low-conic, discoid, dark yellow or purple-brown. Fruit ovoid, brown, 2.0–2.5 × 1.1–1.3 mm; ribs acute, prominent; vittae 3 in each furrow, 6 on commissure (Fig. 8). 2n = 14.

##### Phenology.

Flowering from July to August and fruiting from August to September.

##### Distribution and habit.

Qinghai, Gansu, Ningxia, Sichuan, Xizang. It grows on mountains at elevations of 2700–700 m.

##### Additional specimens examined.

China. **Gansu**: Min County, 02 July 1936, *T.P. Wang 4852* (KUN), 19 August 1937, *T.P. Wang 7535* (WUK), 19 August 1937, *T.P. Wang 7535* (PE), 01 July 1936, *T.P. Wang 4742* (PE), 02 July 1936, *T.P. Wang 4852* (PE), 30 June 1936, *T.P. Wang 4699* (PE); Tianzhu County, *Y.Q. He 4267* (WUK), 12 July 1959, *Y.Q. He 4628* (WUK); Yuzhong County, 09 August 1959, *Y.Q. He 5981* (WUK), 04 August 2000, *X.G. Sun et al. 2126* (PE), 2 September 2023, *L.H. Wang & J.L. Li BNU2023-WLH242* (BNU); Xiahe County, 10 July 1937, *T.P. Wang 7171* (WUK), 29 July 1937, *K.T. Fu 1438* (PE); Minle County, 29 August 1934, *C.W. Yao 336* (NAS); Hezuo County, 09 September 2011, *X. Yin et al. LiuJQ-GN-2011-278* (KUN); Maqu County, 04 August 2011, *X. Yin et al. LiuJQ-GN-2011-280* (KUN); Shandan County, 10 July 1959, *Y.Q. He 4052* (WUK); **Qinghai**: Tianjun County, 05 August 2022, *L.H. Wang et al. BNU2022-WLH030* (BNU); Gangcha County, 30 July 2022, *L.H. Wang et al. BNU2022-WLH017* (BNU); Huzhu County, 05 August 2022, *Q.Y. Zhang ZQY2022003* (BNU); Xunhua County, 06 August 2022, *L.H. Wang et al. BNU2022-WLH039* (BNU), 06 August 2022, *L.H. Wang et al. BNU2022-WLH051* (BNU); Hulong County, 07 August 2022, *LH Wang et al. BNU2022-WLH061* (BNU); Xining City, 11 September 2022, *S.B. Zhang BNU2022-0911* (BNU); Haeyan County, 04 July 1958, *B.Q. Zhong 8410* (KUN); Angqian County, 11 August 1972, *Zangyao team 1283* (KUN); Qilian County, 27 July 1958, *Gan & Qing*, *BQ Zhong 8573* (WUK), 12 August 2013, *X.C. Chen et al. 4487* (HNWP); Xinghai County, 09 August 1919, *T.N. He 416* (WUK); Datong County, 17 August 1962, *Fan & Liang 00495* (HNWP), *B.W. Li 72-070* (HNWP); Ghindu County, 10 August 1983 *X.J. Xun 83-144* (HNWP), 15 August 1996, *T.N. Ho* et al. 1887 (PE), 15 August 1996, *T.N. Ho et al. 1887* (HNWP); Zeku County, 21 August 1967, *L.H. Zhou 1628* (HNWP); Tungrin County, 24 July 1970, *S.W. Liu et al. 1412* (HNWP), 07 August 2010, *S.L. Chen et al. ChenSL0915* (KUN); Menyuan County, 10 July 1970, *L.H. Zhou 1036* (HNWP); Zhidoi County, 10 September 1966, *L.H. Zhou 454* (HNWP); Yushu County, 30 August 1996, *T.N. Ho et al. 2698* (PE), 12 August 1964, *Qinghai Plant Team 620* (WUK); Nangqen County, 05 September 199, *T.N. Ho et al. 2913* (PE); Huangzhong County, 25 July 2014, *Y.H. Wu 050810* (HNWP), 25 July 2014, Y.H. Wu 050644 (HNWP); **Ningxia**: Jingyuan County, 14 August 1942, *T.P. Wang 13561* (KUN); Tongxin County, 12 August 1981, *Y.P. Xu et al. 1701* (WUK); Longde County, 10 July 1942, *T.P. Wang 13052* (WUK); Guyuan County, 07 August 1953, *T.P. Wang 17175* (WUK); **Sichuan**: Ruoergai, County, *X.J. He et al. SCU-20080522* (KUN), 15 July 1993, *Z.M. Tan 93-88* (PE), 06 August 1961, *S. Jiang 6833* (PE); Hongyuan County, 18 September 2012, *Y.D. Gao et al. GaoXF-12-029* (KUN); Pingwu County, *H.L. Tsising 10904* (NAS); Daofu County, 06 September 1960, *Sichuan Team 16217* (NAS); Songpan County, 3 October 1983, *F.D. Pu et al. 021* (CDBI), 02 August 1984, *J. He et al. 140* (CDBI); Daoge County, 01 August 1980, *Vegetation group 28404* (CDBI); Baxoi County, 27 August 1973, *Qinghai-Tibet Team 73-1267* (PE), 14 September 2008, *T. Zhang et al. 08CS701* (KUN), 15 August 2014, *X.C. Chen et al. 032-2* (HNWP), 15 August 2014, *X.C. Chen et al. 032* (HNWP); Wuqi County, 27 August 1976, *Tibet Expedition Team 12951* (PE).

## Supplementary Material

XML Treatment for Bupleurum parvifolium

## ﻿Additional information

### Author ORCIDs

Quan-Ru Liu https://orcid.org/0000-0003-4270-4746

### Data availability

All of the data that support the findings of this study are available in the main text or Supplementary Information.

## ﻿Appendix 1

**Table A1.** GenBank accession numbers of DNA sequences downloaded from NCBI.

Taxa	Accession number	Taxa	Accession number
*B.boissieuanum*	NC036017	*B.longiradiatum*	MT261186
*B.candollei*	MT261183	*B.marginatum*	MN968501
*B.chinense*	MT083933	*B.rockii*	MT261189
*B.densiflorum*	MT261184	*B.shanianum*	MW135452
*B.euphorbioides*	NC053888	*B.sibiricum*	MT261190
*B.falcatum*	NC027834	*B.sikangense*	NC056803
*B.fruticosum*	NC060426	*B.thianschanicum*	MT261192
*B.gibraltaricum*	NC060427	*P.astrantioideum*	NC071798
*B.hamiltonii*	NC056298	*P.szechenyii*	NC071808

## Supplementary materials

Supplementary material 1 Specimens for measurements and initial measurements of traits data Data type xlsx https://binary.pensoft.net/file/1007056This dataset is made available under the Open Database License (http://opendatacommons.org/licenses/odbl/1.0/). The Open Database License (ODbL) is a license agreement intended to allow users to freely share, modify, and use this Dataset while maintaining this same freedom for others, provided that the original source and author(s) are credited.Author: Li-Hua Wang

Supplementary material 2 Traits selected for subsequent principal component and cluster analysis after the first PGA Data type xlsx https://binary.pensoft.net/file/1007057This dataset is made available under the Open Database License (http://opendatacommons.org/licenses/odbl/1.0/). The Open Database License (ODbL) is a license agreement intended to allow users to freely share, modify, and use this Dataset while maintaining this same freedom for others, provided that the original source and author(s) are credited.Author: Li-Hua Wang
